# Evaluation of an Electricity-free, Culture-based Approach for Detecting Typhoidal *Salmonella* Bacteremia during Enteric Fever in a High Burden, Resource-limited Setting

**DOI:** 10.1371/journal.pntd.0002292

**Published:** 2013-06-27

**Authors:** Jason R. Andrews, Krishna G. Prajapati, Elizabeth Eypper, Poojan Shrestha, Mila Shakya, Kamal R. Pathak, Niva Joshi, Priyanka Tiwari, Manisha Risal, Samir Koirala, Abhilasha Karkey, Sabina Dongol, Shawn Wen, Amy B. Smith, Duncan Maru, Buddha Basnyat, Stephen Baker, Jeremy Farrar, Edward T. Ryan, Elizabeth Hohmann, Amit Arjyal

**Affiliations:** 1 Division of Infectious Diseases, Massachusetts General Hospital, Boston, Massachusetts, United States of America; 2 Harvard Medical School, Boston, Massachusetts, United States of America; 3 Oxford University Clinical Research Unit, Patan Hospital, Patan Academy of Health Sciences, Kathmandu, Nepal; 4 D-Lab, Massachusetts Institute of Technology, Cambridge, Massachusetts, United States of America; 5 Department of Medicine, Brigham and Women's Hospital, Boston, Massachusetts, United States of America; 6 The Hospital for Tropical Diseases, Wellcome Trust Major Overseas Programme, Oxford University Clinical Research Unit, Ho Chi Minh City, Vietnam; 7 Centre for Tropical Medicine, Oxford University, Oxford, United Kingdom; 8 The London School of Hygiene and Tropical Medicine, London, United Kingdom; 9 Harvard School of Public Health, Boston, Massachusetts, United States of America; University of California San Diego School of Medicine, United States of America

## Abstract

**Background:**

In many rural areas at risk for enteric fever, there are few data on *Salmonella enterica* serotypes Typhi (*S.* Typhi) and Paratyphi (*S.* Paratyphi) incidence, due to limited laboratory capacity for microbiologic culture. Here, we describe an approach that permits recovery of the causative agents of enteric fever in such settings. This approach involves the use of an electricity-free incubator based upon use of phase-change materials. We compared this against conventional blood culture for detection of typhoidal *Salmonella*.

**Methodology/Principal Findings:**

Three hundred and four patients with undifferentiated fever attending the outpatient and emergency departments of a public hospital in the Kathmandu Valley of Nepal were recruited. Conventional blood culture was compared against an electricity-free culture approach. Blood from 66 (21.7%) patients tested positive for a Gram-negative bacterium by at least one of the two methods. Sixty-five (21.4%) patients tested blood culture positive for *S.* Typhi (30; 9.9%) or *S.* Paratyphi A (35; 11.5%). From the 65 individuals with culture-confirmed enteric fever, 55 (84.6%) were identified by the conventional blood culture and 60 (92.3%) were identified by the experimental method. Median time-to-positivity was 2 days for both procedures. The experimental approach was falsely positive due to probable skin contaminants in 2 of 239 individuals (0.8%). The percentages of positive and negative agreement for diagnosis of enteric fever were 90.9% (95% CI: 80.0%–97.0%) and 96.0% (92.7%–98.1%), respectively. After initial incubation, *Salmonella* isolates could be readily recovered from blood culture bottles maintained at room temperature for six months.

**Conclusions/Significance:**

A simple culture approach based upon a phase-change incubator can be used to isolate agents of enteric fever. This approach could be used as a surveillance tool to assess incidence and drug resistance of the etiologic agents of enteric fever in settings without reliable local access to electricity or local diagnostic microbiology laboratories.

## Author Summary

Every year, 20 million people worldwide suffer from typhoid, a bacterial infection spread by contaminated food and water, and over 200,000 die from the infection. However, few data are available on the prevalence and antimicrobial resistance profiles of the causative agents of typhoid, especially in settings without reliable access to laboratories and electricity. Here, we describe an approach that permits recovery of the causative agents of typhoid that requires no electricity, laboratory infrastructure, or specialized laboratory personnel at the site of patient contact. This approach involves the use of an electricity-free incubator, consisting of an insulated container and reusable packets filled with a chemical that upon warming in hot water or direct sun, maintains 38°C for 24 hours. We used blood culture bottles with a color indicator that signals growth of bacteria and an antibiotic that selects for Gram-negative bacteria. We validated this approach in a clinical study among individuals with fever presenting to a hospital in urban Nepal, demonstrating that the approach performed comparably to conventional blood culture for isolating the bacteria causing typhoid. Such an approach would permit an estimate of the burden of typhoid in areas where such data are lacking, which could inform control strategies.

## Introduction

Enteric fever is a febrile illness caused by *Salmonella enterica* serotype Typhi (*S.* Typhi) or Paratyphi (*S.* Paratyphi) A, B or C. There are an estimated 21.6 million new infections with *S.* Typhi and 5.4 million with *S.* Paratyphi worldwide annually, and over 200,000 deaths [1]. The majority of the disease burden lies in South Asia, where access to accurate diagnostic testing is limited. In rural areas especially, there are very few data on the prevalence of enteric fever and drug resistance among its causative agents. In spite of this, patients presenting to health facilities with fever and no localizing symptoms are frequently presumed to have enteric fever and provided antibiotics directed at this entity. Additionally, with a number of improved vaccines for typhoid in development, there is a need to evaluate the burden of *S.* Typhi and *S.* Paratyphi in rural settings to project the need for and potential impact of new vaccines and other control programs. Further, there are increasing reports of *S.* Typhi and *S.* Paratyphi with reduced susceptibility or overt resistance to fluoroquinolones and azithromycin in South and Southeast Asia [2]–[4]; these antibiotics are among the most widely used in the treatment of individuals with enteric fever. Consequently, improved surveillance for drug susceptibility will be an important component in directing antimicrobial therapy policies to avert morbidity and mortality from enteric fever.

Serologic tests for typhoid have limited sensitivity and specificity in endemic settings and do not provide information on antimicrobial susceptibility. Blood culture and microbiological identification require skilled personnel, specialized laboratory equipment, and an uninterrupted electricity supply. These are not available in many healthcare facilities in low and middle income countries, particularly in rural areas [5]. This has hampered efforts to assess the burden of enteric fever and antimicrobial resistance in many resource-limited settings.

Here, we describe a simple approach for using a phase change-based incubator to isolate Gram-negative bacteria from the blood of patients with undifferentiated fever, noting that this procedure can be performed without electricity, sophisticated equipment, or specialized laboratory personnel. We compared this with conventional culture for recovery of typhoidal *Salmonella*. This approach would allow bacteriologic-based assessments of disease burden in difficult environments and permit recovery of organisms for assessing antimicrobial resistance patterns. Such data could assist with targeted roll out and assessment of typhoid vaccine and control programs in these areas.

## Methods

### Ethics Statement

Approval for this study was obtained from the Institutional Review Board for Human Subjects Research of the Nepal Health Research Council (Kathmandu, Nepal) and the Partners Human Research Committee (Boston, MA, USA). Participants 18 years of age and older were required to provide written informed consent in Nepali for enrollment in this study. For younger patients, parents/guardians provided written informed consent for study purposes after the child verbally assented to have the blood drawn. We followed the Standards for the Reporting of Diagnostic Accuracy Studies (STARD) [6].

### Study Setting and Population

This study was performed between July 2012 and October 2012 at Patan Hospital in the Kathmandu Valley of Nepal. All patients presenting to the outpatient or emergency departments with undifferentiated fever (fever and no alternative diagnosis by history and physical exam) during the study period were eligible for enrollment in the study. Patients with presumptive alternative diagnoses (cellulitis, pneumonia, urinary tract infection) were not included. Children under the age of 2 years and pregnant women were excluded; patients who were receiving antibiotics prior to presentation were not excluded.

### Study Procedures

Demographic and clinical information was obtained from patients who consented to participation in the study. These data included, age, sex, location of residence, clinical symptoms, duration of symptoms, and antimicrobial usage in the week prior to enrollment. An additional 4 ml of blood was collected from study patients as part of the routine venipuncture that was performed for their diagnostic evaluation (complete blood count, biochemical tests, and conventional blood culture).

**Figure 1:**
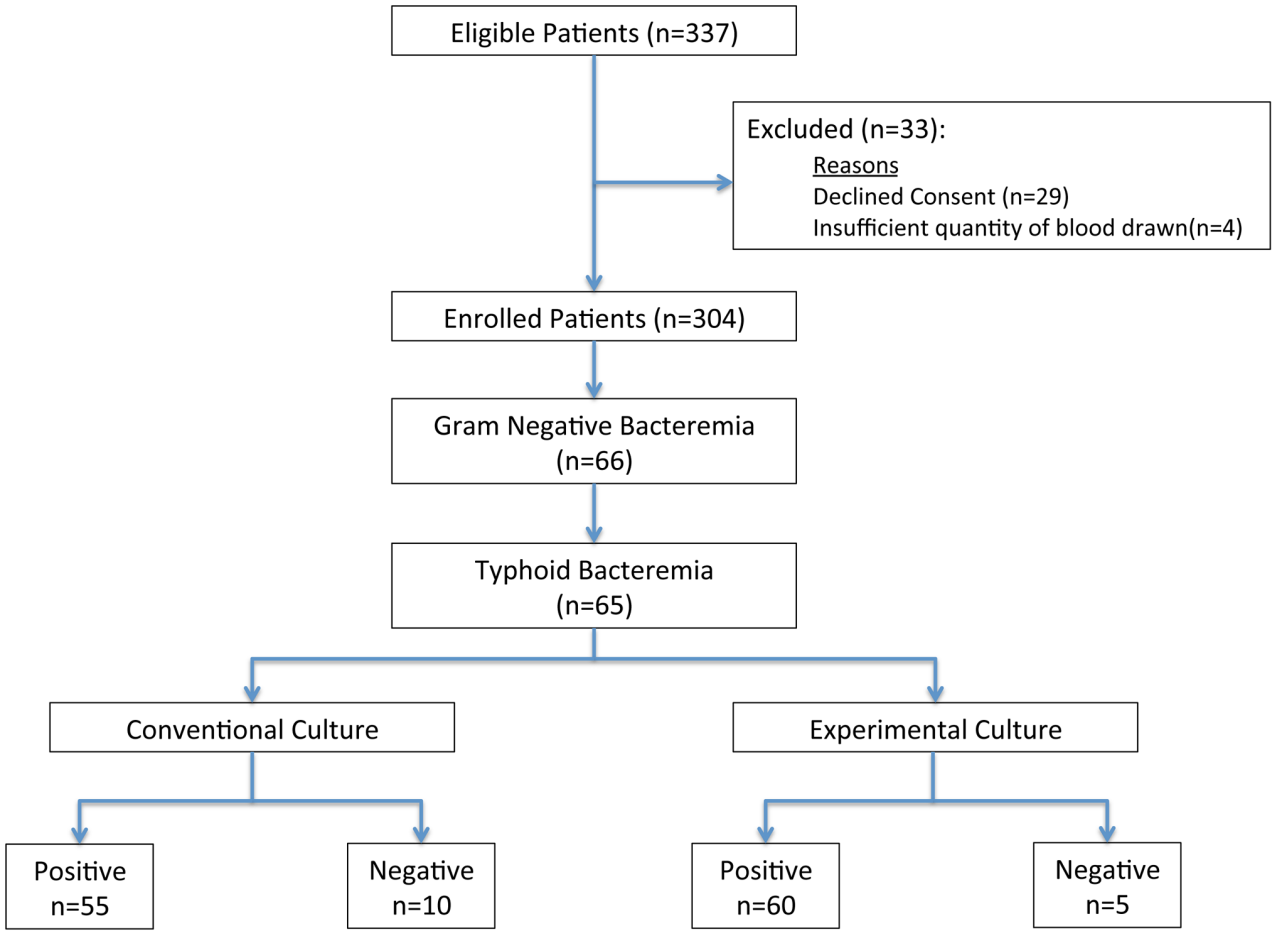
Flow diagram of patients recruited and enrolled in the study.

**Figure 2:**
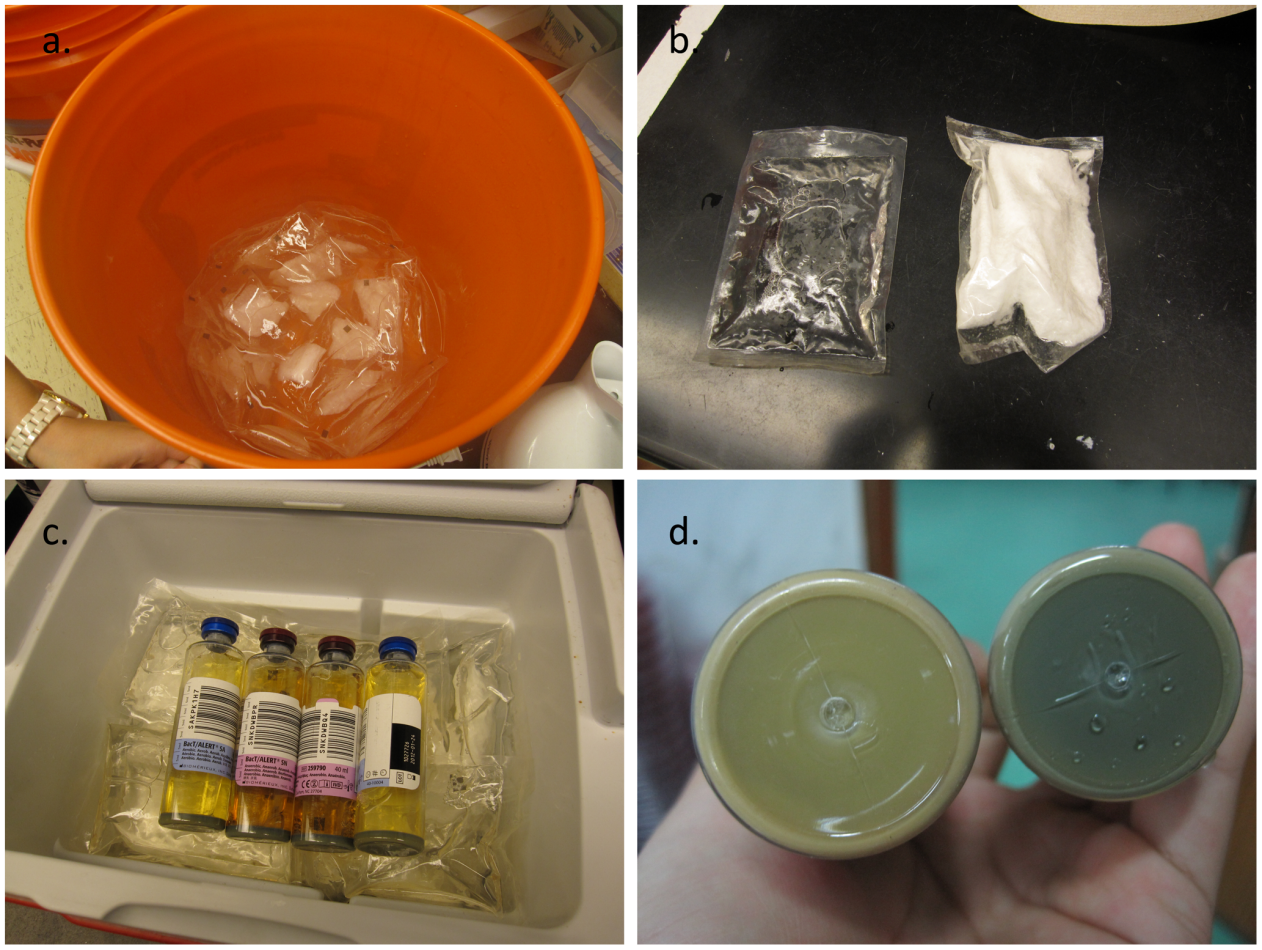
Electricity-free typhoid diagnostic approach. a. packets containing a phase-change material are heated in water or direct sunlight. b. after heating the phase change packet (right), the material appears as a clear liquid (left). c. phase change packets and bottles, containing blood from typhoid suspects and vancomycin additive, are placed into a insulated container. d. bottles are inspected every 24 hours for color change of a CO_2_ detector, which distinguishes positive (left) from negative (right) cultures.

BacT/ALERT (Biomérieux, Durham, NC, USA) blood culture bottles were inoculated with 4 ml of blood and 250 µg of vancomycin hydrochloride to suppress Gram-positive bacteria. Bottles were placed in a Portatherm electricity-free incubator, which maintains a constant temperature using phase-change materials. Fifty sealed packets containing 1-tetradecanol (a material that melts at 38°C) were prepared by submerging them in a hot water bath (Figure 1a). The reusable packets of 1-tetradecanol packets (Figure 1b) maintain a temperature of 38°C while changing phase from liquid back to solid (Figure 2). The packets were placed into an insulated container (vaccine storage boxes, which are low-cost and widely available in developing countries). The blood culture bottles were then placed together with melted phase-change packets into the containers and closed them (Figure 1c).

All blood culture bottles were inspected daily for discoloration of a CO_2_ indicator on the base of the bottle, indicating bacterial growth (Figure 1d). Bottles that demonstrated bacterial growth were removed, and the incubator was reloaded with freshly melted packets; this process was repeated daily for up to seven days. After seven days, subcultures were performed on samples from all negative bottles in both study arms onto Blood, MacConkey and Chocolate agars. Subculture and identification was also performed from all bottles that indicated bacterial growth.

Conventional blood cultures were performed by inoculating 4 ml of blood into 30–50 ml of media containing tryptone soya broth and 0.05% sodium polyanetholesulfonate. Per hospital standard practice, BACTEC PEDS PLUS bottles (Becton Dickinson, Sparks, MD, USA) were used for pediatric patients. Care was taken to ensure that an equal quantity of blood was inoculated into the conventional system as into the experimental one. Bottles were incubated at 37°C in a standard electric microbiological incubator. Bottles were treated as before. Isolates originating from both methods were identified using standard biochemical tests and serotype-specific antisera (Murex Biotech, Dartford, UK). Both the conventional and experimental culture procedures were overseen by experienced medical microbiologists. Laboratory personnel examining blood culture bottles from each method were blind to the results of the alternative method.

To evaluate how long after incubation *S.* Typhi and *S.* Paratyphi could be isolated from the blood culture bottles, we inoculated bottles with 1 colony forming unit/ml and 5 ml of whole blood, incubated them for a week, and then kept them at room temperature, performing subculture on a weekly basis.

### Statistical Analyses

The primary outcome was the proportion of cultures positive for *S.* Typhi or *S.* Paratyphi A in the conventional and experimental culture methods as a proportion of the total patients enrolled with undifferentiated fever. Because false positive results for isolation and identification of typhoid using biochemical testing and antisera would be unlikely by either culture system, the proportion that were positive by each system, using the total number of positive samples by either system as the denominator, was also calculated. Conventional blood culture with a sensitivity of around 40–60% is an imperfect reference standard; therefore the percentage positive and negative agreement rather than sensitivity and specificity are reported, consistent with expert recommendations and guidance of the United States Food and Drug Administration [7]. Point estimates together with exact 95% binomial confidence intervals for percent agreement are reported. Furthermore, the proportion of blood cultures that were positive among individuals receiving antimicrobials within the past week was compared with the proportion among those who had not received antimicrobials by Fisher's Exact Test. Time to positivity of culture was compared by the Wilcoxon Signed Rank Test.

## Results

**Figure 3:**
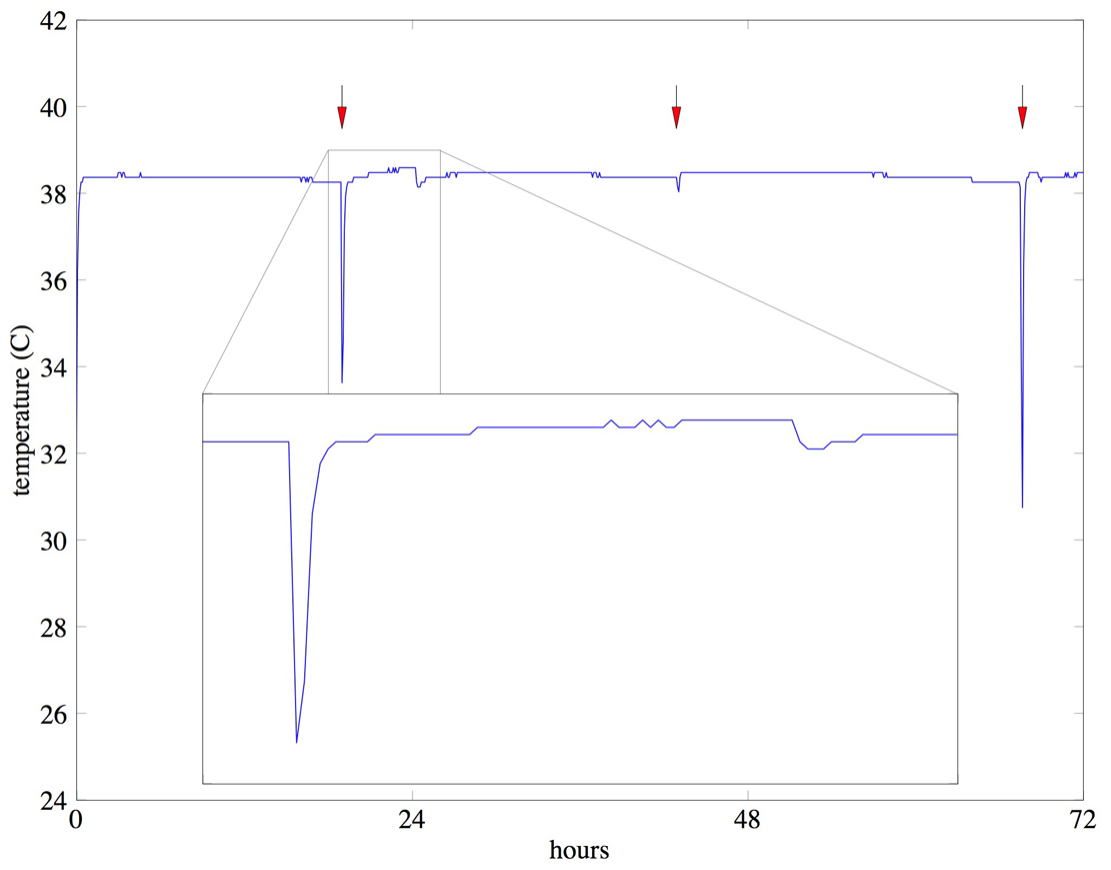
Sample temperature readings from phase-change incubator. Example of temperature readings, recorded every five minutes inside the incubator over a period of 72 hours. Exchange of phase change packets was performed daily (red arrows) to “recharge” the incubator and leads to brief periods of lower temperatures followed by higher temperatures (inset), which were not found to impact bacterial growth.

Three hundred and thirty-seven patients with undifferentiated fever were approached for participation in this study, of whom 308 (91.4%) consented to participate in the study (Figure 3). An inadequate quantity of blood was drawn to perform both tests in 4 patients; the final enrolled population was 304 patients. The mean age of study participants was 16 years (IQR: 9–25 years) and 41.1% were female. The median duration of fever prior to enrollment was 5 days (IQR: 4–6 days).

**Table 1:** Bacteria isolated among participants in the study according to culture method (either, conventional, or experimental).

	Either Method		Conventional		Experimental	
Bacteria Isolated	n (%)		n (%)		n (%)	
Enteric Pathogens	65	(21.4)	55	(18.1)	60	(19.7)
*S.* Paratyphi A	35	(11.5)	29	(9.5)	31	(10.2)
*S.* Typhi	30	(9.9)	26	(8.6)	29	(9.5)
Other Pathogens	5	(1.6)	5	(1.6)	0	(0)
*Streptococcus pneumoniae*	1	(0.3)	1	(0.3)	0	(0)
Other *Streptococci spp.*	2	(0.7)	2	(0.7)	0	(0)
*Staphylococcus aureus*	1	(0.3)	1	(0.3)	0	(0)
*Acinetobacter spp.*	1	(0.3)	1	(0.3)	0	(0)
Commensal Organisms	23	(7.6)	21	(6.9)	2	(0.7)
Coagulase-negative *Staphylococcus*	16	(5.3)	14	(4.6)	2	(0.7)
*Bacillus spp.*	5	(1.6)	5	(1.7)	0	(0)
*Micrococcus spp.*	2	(0.7)	2	(0.7)	0	(0)
No Bacteria Isolated	211	(69.4)	223	(73.4)	242	(79.6)

**Table 2:** Culture-confirmed diagnosis of enteric fever by conventional blood culture in comparison with results from the experimental procedure.

		Conventional Blood Culture		
		Positive	Negative	Total
Experimental	Positive	50	10*	60
Blood Culture	Negative	5	239	244
	Total	55	249	304

* The experimental blood culture was positive in two additional patients that were negative by conventional blood culture, but the organisms were probable skin contaminants. These are captured in percent agreement calculations.

Other pathogens were identified in blood from 5 of 304 (1.6%) of patients by the conventional blood culture system; we did not identify an alternative pathogen using the experimental blood culture. The alternative pathogens included *Streptococcus pneumoniae* (1 patient), other *Streptococci* (2 patients), *Staphylococcus aureus* (1 patient) and *Acinetobacter spp* (1 patient). Twenty-three patients (7.6%) had blood cultures positive for organisms believed to be commensal or skin contaminants; these were seen in 21 (6.9% of total patients) of the conventional blood cultures and 2 (0.7%) of the experimental blood cultures. The majority of commensal organisms (16 of 23; 69.6%) were coagulase-negative *Staphylococci*.

Accounting for the two “false-positive” cases of skin flora in the experimental procedure that would not have been distinguished from typhoid (without subculture), the overall percent agreement between the experimental blood culture and the conventional blood culture for diagnosis of enteric fever was 94.4% (95% CI: 91.2%–96.7%). The percent positive agreement was 90.9% (95% CI: 80.0%–97.0%) and the percent negative agreement was 95.2% (95% CI: 91.7%–97.5%).

The median time to positivity for typhoid isolates was 2 days for both the conventional blood culture (IQR: 1–3 days; range: 1–4 days) and the experimental blood culture (IQR: 2-2 days; range: 1–5 days). The positive culture result was available at least one day earlier by the conventional culture procedure in 14 patients, by at least one day earlier by the experimental culture in 9 patients, and on the same day in 27 patients (p = 0.38).

Eighty-two patients (27.3%) had received an antimicrobial within the previous week. The median duration of antimicrobial use was 3 days (IQR: 2–5 days). The percentage of patients with positive cultures was higher among individuals receiving antibiotics (30.5%) compared with those not receiving antibiotics (18.0%) (p = 0.027). Antibiotic exposure did not impact percent agreement between the conventional and experimental systems.

### Duration of Bacterial Viability

After incubation for a week, *S.* Typhi and *S.* Paratyphi A remained viable at room temperature, as demonstrated by subculture, for at least six months without supplementation of additional media.

## Discussion

Global estimates of the burden of typhoid are derived primarily from studies in dense urban areas, where culture microbiology is available. In rural areas, where a large proportion of the population resides, there are very few data on the incidence of typhoid due to lack of laboratory capacity. Because typhoid cannot be reliably distinguished from other febrile illnesses—such as viral infections, leptospirosis, and rickettsial infections [8]–[11], syndrome-based surveillance is inadequate. Last year, more than 500,000 cases of typhoid were reported in the public sector alone in Nepal, for an incidence of 1.9 episodes per 100 person-years [12]. This is an order of magnitude higher than the incidence estimated through surveys and other forms of surveillance in high-burden, urban settings [13], [14]. However, the majority of the diagnoses reported in Nepal were made empirically in locations without diagnostic laboratory capacity and may not be accurate. Despite its limited sensitivity, blood culture remains the best available method for establishing a diagnosis of typhoid and the only currently used means for assessing antimicrobial resistance, but it is not available in many high burden settings due to lack of reliable electricity, laboratory infrastructure and trained personnel [5], [15]. As a result, there are few data on the burden of *S.* Typhi and *S.* Paratyphi in areas without these resources, especially rural areas and even more limited data on resistance to commonly used antimicrobials in these settings.

Here we describe a simple approach to recovering Gram-negative organisms from blood that is not dependent upon electricity or sophisticated laboratory infrastructure at the site of medical care and can be performed by health personnel with minimal training. This procedure only requires drawing blood, placing the blood into a blood culture bottle containing vancomycin and capable of a colorimetric change, and then incubating the bottle in an insulated container into which reusable, heated packets containing 1-tetradecanol have been placed. We found that this method had comparable yield to conventional blood culture in recovering *S.* Typhi and *S.* Paratyphi A. By collecting positive bottles and performing identification and susceptibility testing at reference laboratories, this approach could enable ongoing surveillance for enteric fever prevalence among febrile patients and for antimicrobial sensitivity profiles in rural settings and other settings lacking adequate resources. Of note, as part of this project, we demonstrated that typhoidal *Salmonella* remain viable in the bottles for at least six months at room temperature after sample collection. Consequently, bottles could be collected from rural sites on a periodic basis for identification and susceptibility testing.

Multiple studies in South and Southeast Asia have demonstrated that *S.* Typhi and *S.* Paratyphi account for 90–100% of Gram-negative bacteria isolated from blood among patients presenting to hospitals [8], [16]–[24]. This may be particularly true in children, in whom other etiologies of Gram-negative bacteremia are less common in many resource-limited areas. In our study, 65 of 66 (98.5%) Gram-negative bacteremia diagnoses were attributable to *S.* Typhi or *S.* Paratyphi A. However, these studies were performed in an urban setting, and there are few data from rural health centers. By determining surveillance data on the prevalence of enteric fever and drug resistance in rural settings among patients with acute febrile illnesses, practitioners in those settings may be better able to make decisions about antimicrobial use for patients presenting with undifferentiated fever. The limited sensitivity of this culture approach does complicate estimation of prevalence of enteric fever; however, a number of statistical methods have been developed to estimate disease prevalence in the context of imperfect test accuracy [25]. The excellent specificity of culture, in contrast to serologic approaches, makes such estimates relatively straightforward.

Because most Gram-positive organisms isolated in blood cultures in many resource-limited areas are skin contaminants, we added an antibiotic to suppress Gram-positive bacteria, focusing our evaluation on Gram-negative organisms. In the conventional blood culture arm, in which this antibiotic was not added, Gram-positive pathogens only accounted for 6.7% of all pathogenic bacteria isolated. The majority (84%) of Gram-positive bacteria isolated were thought to be commensal skin contaminants. Of note, the antibiotic may be eliminated if surveillance for Gram-positive organisms is of interest in the particular setting being evaluated.

Across Nepal, electricity interruptions or scheduled rationing are common [26]. Even urban areas, such as Kathmandu, face 5 hours of scheduled outages per day during the wet season and up to 18 hours a day of scheduled outages per day during the dry season, with the outages projected to continue to increase for the foreseeable future [26]. Health centers are typically not exempted from these outages. In rural areas, outages can be unpredictable and long term, sometimes lasting for months. Throughout the developing world, maintaining a stable and reliable electricity supply is a common challenge for rural health centers, and this poses a challenge to supporting even basic microbiology laboratories. The phase change-based incubation method we describe has already been used for performing interferon gamma release assays for tuberculosis in resource-limited settings, and could have an array of uses in such areas [27].

In comparing diagnostic procedures with an imperfect reference standard, there has been debate about the appropriate characteristics to report and statistical tests to use for comparisons. In the case of typhoid, multiple studies have demonstrated that the sensitivity of blood culture is only 40–60% [28]–[32]. In cases of an imperfect reference standard, many expert bodies, including the United States Food and Drug Administration, advise against reporting sensitivity, specificity and predictive values. Instead, as we did here, guidelines recommend reporting the percent positive and negative agreement [7]. We chose to also report the proportion of cases identified by each diagnostic approach among all culture-confirmed cases identified by either diagnostic. We believe this approach is valid and useful because *Salmonella* isolated from either culture system are unlikely to be false positives in the absence of laboratory contamination. We used blood cultures results as our sole diagnostic criteria rather than clinical or serologic criteria because the latter are not yet well established in this setting, and the primary purpose of this study was to evaluate an alternative to conventional cultures for resource-limited settings. While the experimental system identified 10 cases of typhoid not identified by the conventional system (and the conventional system identified 5 cases not identified by the experimental system), these differences were not statistically significant and are consistent with concordance rates seen for paired conventional blood cultures [33].

This study and procedure have several important limitations. First, this procedure focused on recovery of the etiologic agents of enteric fever; other important bacterial pathogens, such as *Brucella* spp. and fastidious organisms, may be more difficult to isolate with this method [34], [35]. However, many widely used diagnostic tests focus on a single pathogen, including serologic tests for typhoid, brucellosis, Q fever, and leptospirosis; smear or rapid diagnostic tests for malaria or tuberculosis; and PCR techniques for influenza and dengue. Future work could examine the recovery of other bacterial pathogens.

We used only 4 ml of blood for culture, due to local practices shaped by patient concerns about phlebotomy of larger blood volumes, particularly among children. Reller and colleagues utilized a median blood volume of 1.96 ml when investigating pediatric patients for typhoid in Karachi, Pakistan and found all isolates were recovered from <5 ml of blood [19]. Because *S.* Typhi and *S.* Paratyphi are often present in low quantities in the blood [36], the culture of larger blood volumes (up to 15 ml) may increase the yield [37], though some studies have failed to demonstrate this [32]. A number of features beyond blood volume and low organisms load lessen the likelihood of recovering viable bacteria in blood, including late presentation and prior use of antibiotics. The presence of sodium polyanetholesulfonate in both the conventional and experimental blood cultures is thought to improve culture yield [38]. As we work to design a reusable bottle that can be produced on site, ox bile broth may serve the same purpose, by lysing blood cells that inhibit bacterial growth [37], [39]–[41]. Ox bile broth has the added feature of inhibiting the growth of many other bacteria including skin flora. The use of vancomycin was designed to suppress Gram-positive organisms, which more often than not represent contaminants (23 of 27 Gram positive isolates in this study were probable contaminants from the skin). However, 4 of the 304 patients screened had Gram-positive bacteremia with pathogenic organisms, which would be missed by the experimental approach. These data demonstrate that the experimental system is not ideal for a setting with more sophisticated laboratory capabilities. Simple chromogenic approaches to distinguish Gram positive and negative isolates in a closed culture system would add value to this procedure while maintaining simplicity for use by non-laboratory personnel.

The one-time cost of the incubator and reusable phase change packets is approximately $50, and the per-use costs of the blood culture bottles and additive were approximately $2.30. This per-use cost is substantially lower than some newer generation serologic tests for typhoid [42]. However, these costs are still high for Nepal, where annual *per capita* health expenditure is $30. We are currently working to design a lower cost bottle with a colorimetric growth indicator. Additionally, by performing the test on a random sample of patients presenting with fever, as often done in surveillance studies, costs may be minimized. When used for surveillance, subculture and identification costs will accrue for positive bottles; the use of vancomycin averts the costs of identification of skin contaminants. Finally, the technology for the phase change incubator, developed by one of the authors (ABS), is not patented and the phase-change packets could be locally produced.

Reliable, rapid, point-of-care diagnostics for enteric fever and drug resistance are desperately needed in resource-limited settings. While we await such developments, methods for evaluating the prevalence and drug resistance among *S.* Typhi and *S.* Paratyphi in regions for which we have no data would be a significant advance. The approach we described may begin to address this. Further studies to evaluate the challenges of implementing this approach in routine clinical environments in rural settings are needed.

## Supporting Information

Checklist S1 **STARD Checklist.**
(DOC)

## References

[1] Crump JA, Luby SP, Mintz ED (2004) The global burden of typhoid fever. Bull World Health Organ 82: 346–353. 15298225 PMC2622843

[2] Parry CM, Vinh H, Chinh NT, Wain J, Campbell JI, et al (2011) The influence of reduced susceptibility to fluoroquinolones in Salmonella enterica serovar Typhi on the clinical response to ofloxacin therapy. PLoS Negl Trop Dis 5: e1163 doi:10.1371/journal.pntd.0001163 21713025 10.1371/journal.pntd.0001163 PMC3119645

[3] Koirala KD, Thanh DP, Thapa SD, Arjyal A, Karkey A, et al (2012) Highly Resistant Salmonella enterica Serovar Typhi with a Novel gyrA Mutation Raises Questions about the Long-Term Efficacy of Older Fluoroquinolones for Treating Typhoid Fever. Antimicrob Agents Chemother 56: 2761–2762 doi:10.1128/AAC.06414-11 22371897 10.1128/AAC.06414-11 PMC3346606

[4] Vlieghe ER, Phe T, De Smet B, Veng CH, Kham C, et al (2012) Azithromycin and ciprofloxacin resistance in Salmonella bloodstream infections in Cambodian adults. PLoS Negl Trop Dis 6: e1933 doi:10.1371/journal.pntd.0001933 23272255 10.1371/journal.pntd.0001933 PMC3521708

[5] Archibald LK, Reller LB (2001) Clinical microbiology in developing countries. Emerging Infect Dis 7: 302–305. 11294729 10.3201/eid0702.010232 PMC2631738

[6] Bossuyt PM, Reitsma JB, Bruns DE, Gatsonis CA, Glasziou PP, et al (2003) Toward complete and accurate reporting of studies of diagnostic accuracy. The STARD initiative. Am J Clin Pathol 119: 18–22 doi:10.1309/8EXC-CM6Y-R1TH-UBAF 12520693 10.1309/8EXC-CM6Y-R1TH-UBAF

[7] United States Department of Health and Human Services, Food and Drug Administration (2007) Statistical Guidance on Reporting Results from Studies Evaluating Diagnostic Tests. Rockville, MD.Available: http://www.fda.gov/MedicalDevices/DeviceRegulationandGuidance/GuidanceDocuments/ucm071148.htm. Accessed 5 December 2012.

[8] Murdoch DR, Woods CW, Zimmerman MD, Dull PM, Belbase RH, et al (2004) The etiology of febrile illness in adults presenting to Patan hospital in Kathmandu, Nepal. Am J Trop Med Hyg 70: 670–675. 15211012

[9] Ross IN, Abraham T (1987) Predicting enteric fever without bacteriological culture results. Trans R Soc Trop Med Hyg 81: 374–377. 3686631 10.1016/0035-9203(87)90139-8

[10] Vollaard AM, Ali S, Widjaja S, Asten HAGH van, Visser LG, et al (2005) Identification of typhoid fever and paratyphoid fever cases at presentation in outpatient clinics in Jakarta, Indonesia. Trans R Soc Trop Med Hyg 99: 440–450 doi:10.1016/j.trstmh.2004.09.012 15837356 10.1016/j.trstmh.2004.09.012

[11] Hosoglu S, Geyik MF, Akalin S, Ayaz C, Kokoglu OF, et al (2006) A simple validated prediction rule to diagnose typhoid fever in Turkey. Trans R Soc Trop Med Hyg 100: 1068–1074 doi:10.1016/j.trstmh.2005.12.007 16697432 10.1016/j.trstmh.2005.12.007

[12] Department of Health Services (2011) Annual Report: 2067/68 (2010/2011). Kathmandu, Nepal: Government of Nepal, Ministry of Health and Population.

[13] Ochiai RL, Acosta CJ, Danovaro-Holliday MC, Baiqing D, Bhattacharya SK, et al (2008) A study of typhoid fever in five Asian countries: disease burden and implications for controls. Bull World Health Organ 86: 260–268 doi:10.2471/BLT.06.039818 18438514 10.2471/BLT.06.039818 PMC2647431

[14] Naheed A, Ram PK, Brooks WA, Hossain MA, Parsons MB, et al (2010) Burden of typhoid and paratyphoid fever in a densely populated urban community, Dhaka, Bangladesh. Int J Infect Dis 14 Suppl 3: e93–99 doi:10.1016/j.ijid.2009.11.023 20236850 10.1016/j.ijid.2009.11.023

[15] Baker S, Favorov M, Dougan G (2010) Searching for the elusive typhoid diagnostic. BMC Infect Dis 10: 45 doi:10.1186/1471-2334-10-45 20205702 10.1186/1471-2334-10-45 PMC2846943

[16] Amatya NM, Shrestha B, Lekhak B (2007) Etiological agents of bacteraemia and antibiotic susceptibility pattern in Kathmandu Model Hospital. JNMA J Nepal Med Assoc 46: 112–118. 18274566

[17] Sharma NP, Peacock SJ, Phumratanaprapin W, Day N, White N, et al (2006) A hospital-based study of bloodstream infections in febrile patients in Dhulikhel Hospital Kathmandu University Teaching Hospital, Nepal. Southeast Asian J Trop Med Public Health 37: 351–356. 17124998

[18] Pradhan R, Shrestha U, Gautam SC, Thorson S, Shrestha K, et al (2012) Bloodstream Infection among Children Presenting to a General Hospital Outpatient Clinic in Urban Nepal. PLoS ONE 7: e47531 doi:10.1371/journal.pone.0047531 23115652 10.1371/journal.pone.0047531 PMC3480362

[19] Reller ME, Zaidi AKM, Sultana S, Azeem S, Hanif B, et al (2009) Controlled evaluation of Bactec Peds Plus/F and Bactec lytic/10 anaerobic/F media for isolation of Salmonella enterica serovars typhi and paratyphi A from blood. J Clin Microbiol 47: 245–246 doi:10.1128/JCM.01452-08 19005140 10.1128/JCM.01452-08 PMC2620831

[20] Brooks WA, Hossain A, Goswami D, Nahar K, Alam K, et al (2005) Bacteremic typhoid fever in children in an urban slum, Bangladesh. Emerging Infect Dis 11: 326–329 doi:10.3201/eid1102.040422 15752457 10.3201/eid1102.040422 PMC3320465

[21] Narayanappa D, Sripathi R, Jagdishkumar K, Rajani HS (2010) Comparative study of dot enzyme immunoassay (Typhidot-M) and Widal test in the diagnosis of typhoid fever. Indian Pediatr 47: 331–333. 19430063 10.1007/s13312-010-0062-x

[22] Gasem MH, Smits HL, Goris MGA, Dolmans WMV (2002) Evaluation of a simple and rapid dipstick assay for the diagnosis of typhoid fever in Indonesia. J Med Microbiol 51: 173–177. 11865843 10.1099/0022-1317-51-2-173

[23] Jesudason MV, Sivakumar S (2006) Prospective evaluation of a rapid diagnostic test Typhidot for typhoid fever. Indian J Med Res 123: 513–516. 16783041

[24] Kawano RL, Leano SA, Agdamag DMA (2007) Comparison of serological test kits for diagnosis of typhoid fever in the Philippines. J Clin Microbiol 45: 246–247 doi:10.1128/JCM.01403-06 17065261 10.1128/JCM.01403-06 PMC1828988

[25] Walter SD, Irwig LM (1988) Estimation of test error rates, disease prevalence and relative risk from misclassified data: a review. J Clin Epidemiol 41: 923–937. 3054000 10.1016/0895-4356(88)90110-2

[26] Ratna Sansar S (2010) Electricity crisis (load shedding) in Nepal, its manifestations and ramifications. Hydro Nepal 7–17.

[27] Dominguez M, Smith A, Luna G, Brady MF, Austin-Breneman J, et al (2010) The MIT D-lab electricity-free PortaTherm™ incubator for remote testing with the QuantiFERON®-TB Gold In-Tube assay. Int J Tuberc Lung Dis 14: 1468–1474. 20937189 PMC3111905

[28] Gilman RH, Terminel M, Levine MM, Hernandez-Mendoza P, Hornick RB (1975) Relative efficacy of blood, urine, rectal swab, bone-marrow, and rose-spot cultures for recovery of Salmonella typhi in typhoid fever. Lancet 1: 1211–1213. 48834 10.1016/s0140-6736(75)92194-7

[29] Guerra-Caceres JG, Gotuzzo-Herencia E, Crosby-Dagnino E, Miro-Quesada M, Carrillo-Parodi C (1979) Diagnostic value of bone marrow culture in typhoid fever. Trans R Soc Trop Med Hyg 73: 680–683. 538809 10.1016/0035-9203(79)90020-8

[30] Vallenas C, Hernandez H, Kay B, Black R, Gotuzzo E (1985) Efficacy of bone marrow, blood, stool and duodenal contents cultures for bacteriologic confirmation of typhoid fever in children. Pediatr Infect Dis 4: 496–498. 3900945 10.1097/00006454-198509000-00011

[31] Hoffman SL, Edman DC, Punjabi NH, Lesmana M, Cholid A, et al (1986) Bone marrow aspirate culture superior to streptokinase clot culture and 8 ml 1∶10 blood-to-broth ratio blood culture for diagnosis of typhoid fever. Am J Trop Med Hyg 35: 836–839. 3089041 10.4269/ajtmh.1986.35.836

[32] Gasem MH, Dolmans WM, Isbandrio BB, Wahyono H, Keuter M, et al (1995) Culture of Salmonella typhi and Salmonella paratyphi from blood and bone marrow in suspected typhoid fever. Trop Geogr Med 47: 164–167. 8560588

[33] Plorde JJ, Tenover FC, Carlson LG (1985) Specimen volume versus yield in the BACTEC blood culture system. J Clin Microbiol 22: 292–295. 3897271 10.1128/jcm.22.2.292-295.1985 PMC268378

[34] Ozkurt Z, Erol S, Tasyaran MA, Kaya A (2002) Detection of Brucella melitensis by the BacT/Alert automated system and Brucella broth culture. Clin Microbiol Infect 8: 749–752. 12445015 10.1046/j.1469-0691.2002.00471.x

[35] Baysallar M, Aydogan H, Kilic A, Kucukkaraaslan A, Senses Z, et al (2006) Evaluation of the BacT/ALERT and BACTEC 9240 automated blood culture systems for growth time of Brucella species in a Turkish tertiary hospital. Med Sci Monit 12: BR235–238. 16810129

[36] Wain J, Diep TS, Ho VA, Walsh AM, Nguyen TT, et al (1998) Quantitation of bacteria in blood of typhoid fever patients and relationship between counts and clinical features, transmissibility, and antibiotic resistance. J Clin Microbiol 36: 1683–1687. 9620400 10.1128/jcm.36.6.1683-1687.1998 PMC104900

[37] Wain J, Diep TS, Bay PVB, Walsh AL, Vinh H, et al (2008) Specimens and culture media for the laboratory diagnosis of typhoid fever. J Infect Dev Ctries 2: 469–474. 19745526 10.3855/jidc.164

[38] Belding ME, Klebanoff SJ (1972) Effect of Sodium Polyanetholesulfonate on Antimicrobial Systems in Blood. Appl Microbiol 24: 691–698. 4640735 10.1128/am.24.5.691-698.1972 PMC380646

[39] Cummins SL (1911) The Anti-Bactericidal Action of the Bile Salts. Epidemiology & Infection 11: 373–380 doi:10.1017/S002217240001682X 10.1017/s002217240001682x PMC2167260 20474462

[40] Kaye D, Palmieri M, Eyckmans L, Rocha H, Hook EW (1966) Comparison of bile and trypticase soy broth for isolation Salmonella from blood. Am J Clin Pathol 46: 408–410. 5921419 10.1093/ajcp/46.3_ts.408

[41] Escamilla J, Santiago LT, Sangalang RP, Ranoa CP, Cross JH (1984) Comparative study of three blood culture systems for isolation of enteric fever Salmonella. Southeast Asian J Trop Med Public Health 15: 161–166. 6095460

[42] Fadeel MA, House BL, Wasfy MM, Klena JD, Habashy EE, et al (2011) Evaluation of a newly developed ELISA against Widal, TUBEX-TF and Typhidot for typhoid fever surveillance. J Infect Dev Ctries 5: 169–175. 21444985 10.3855/jidc.1339

